# Dermatology Resident Basic Exam Scores Are Correlated With American Board of Dermatology Certification Examination Outcomes in the New Certification Model

**DOI:** 10.7759/cureus.113673

**Published:** 2026-07-30

**Authors:** Gillian Heinecke, Ruizhi Huang, Nicole Burkemper, Erik J Stratman

**Affiliations:** 1 Department of Dermatology, SLUCare, SSM Health Saint Louis University, St. Louis, USA; 2 Advanced HEAlth Data (AHEAD) Institute, Saint Louis University School of Medicine, St. Louis, USA; 3 Department of Dermatology, Marshfield Clinic Region - Sanford Health, Marshfield, USA

**Keywords:** board examination, dermatology residency, in-training examination, residency outcome, resident performance

## Abstract

Background

The format of the American Board of Dermatology (ABD) exams has now changed with a self-assessment Basic Exam in the first year of dermatology residency, followed by certification Core Exams during residency and the Applied Exam after completion of residency.

Objective

To determine if the Basic Exam scores correlate with performance on the new certification exams in dermatology.

Methods

Deidentified examination data from residents between 2018 and 2023 was obtained from the ABD. The basic exam scores between those who passed and failed the certification exams were analyzed. The optimal threshold for Basic Exam scores was then calculated using a receiver operating characteristic curve.

Results

For the 1585 residents included, there was a statistically significant difference in Basic Exam performance for those who passed and failed the certification exams (p < 0.001). The threshold cutoff varied by certification exam with a total Basic Exam score range of 75.55 to 79.55 and percentile rank range of 22%-38%.

Limitations

Demographic data was not available to include in the analysis.

Conclusion

The Basic Exam performance correlates with certification exam performance. The average threshold of total score of 78.5 and percentile rank of 31.4% would capture 77% of residents who failed the certification exams.

## Introduction

The format of the American Board of Dermatology (ABD) resident self-assessment and certification examinations has recently changed. Prior to 2018, dermatology residents took an In-Service Training Examination (ITE) each spring of their residency and then sat for a single written certification examination at the end of their residency. The ITE exam was designed to mimic the certification exam and assess residents’ preparedness throughout residency. Evaluation of residents’ ITE and certification examination outcomes in multiple other medical specialties, including Plastic Surgery, Otolaryngology, and Internal Medicine, found that the ITE exam scores are predictive of performance on the written certification exam [1-3]. Further studies in General Surgery found that either voluntary or mandatory remediation for residents with low ITE scores improved their future performance on subsequent ITE exams or increased their likelihood of passing their certification exam on the first attempt for those residents who completed the remediation [4,5].

Since 2018, there has been a single self-assessment exam called the Basic Exam in the spring of a resident's first year of dermatology residency. The Basic Exam is only given to first-year residents and is designed to identify early struggling residents. It is not designed to mimic the board exam, unlike the ITE. This is then followed by the certification examinations starting during residency with four Core Exams followed by the Applied Exam after graduation. The Core Exams consist of four subject-specific exams in medical dermatology, pediatric dermatology, surgical dermatology, and dermatopathology, and can be taken during four different administration sessions starting in late winter in the second year of residency and with three additional sessions during the third year of residency. All Core Exams must be passed prior to being eligible to sit for the Applied Exam [6].

While failure of the certification exam pathway in dermatology is low [7], failure of the exams can have a major impact on both residents and their residency training programs. For the resident, failure can create significant stress during residency, delay initial board certification, and possibly impact their ability to obtain employment following graduation. For residency programs, a low board pass rate can impact ACGME accreditation status, as programs are expected to have either an aggregate pass rate higher than the bottom fifth percentile or an 80% minimum absolute pass rate [8]. Additionally, residents failing their certification exams can negatively affect the culture and the recruitment competitiveness of a residency program.

To date, no studies have investigated the predictive value of the new Basic Exam score on certification exam outcomes, nor does the literature contain guidance on how residents and their residency programs should use the Basic Exam results to guide resident learning to help prepare for subsequent certification exams. The goal of this study was to analyze Basic Exam scores as a predictor for success on the American Board of Dermatology Core and Applied certification exams.

## Materials and methods

The institutional review board at Saint Louis University determined this study to be exempt. The deidentified Basic Exam, Core Exam, and Applied Exam scores from all residents between 2018 and 2023 were gathered from the American Board of Dermatology database. Data collected from the Basic Exam included total score, percentile rank, and sub-scores in visual recognition, medical/general dermatology, pediatric dermatology, dermatopathology, surgical dermatology, and science. The data collected from the Core and Applied Exams included date administered, pass/fail outcome, percentile rank, and reported score. Residents were included in analysis if they took all exams, the Basic Exam, four Core Exams and the Applied Exam. All outcome analyses were based on first-attempt pass/fail outcomes for each Core Exam and the Applied Exam. Residents who took the Basic Exam more than once were excluded. Demographic data was not available.

Summary statistics of Basic Exam score descriptions and Core Exam and Applied Exam Pass/Fail Descriptions were calculated. The Basic Exam component scores were compared between the failure and pass groups for each of the Core and Applied Exams using the Mann-Whitney U test.

Multivariate logistic regression analyses were conducted to assess associations between each Core and Applied Exam Score and the individual Basic Exam component scores. The total score was not included in the multivariable models to avoid redundancy with component subscores. The outcome was defined as CORE and Applied exam performance, with pass coded as 1 and failure coded as 0. Multicollinearity among Basic Exam component scores was assessed using the variance inflation factor (VIF). To further evaluate model stability in the presence of correlated predictors, a penalized (ridge) logistic regression as a sensitivity analysis was conducted.

To evaluate thresholds in Basic Exam total score and percentile rank predictive of Core or Applied Exam passing on first attempt, a receiver operating characteristic (ROC) curve was generated. The curve plotted the True Positive Rate (TPR) against the False Positive Rate (FPR). The optimal threshold was calculated as the point on the ROC curve that maximizes the difference between TPR and FPR (Youden index). Kruskal-Wallis test was also used to assess differences in Basic Exam total scores across different residency programs. Statistical significance was set at p < 0.05. All outcome analyses were conducted with RStudio (Posit Software, Boston, MA, USA).

## Results

Data was analyzed from 1585 residents who had participated in all exams and took the Basic Exam only once. We originally had 1647 residents in our dataset. Two were excluded as they took the Basic Exam twice. Twenty were excluded as they took the Basic but no certification exams. Twenty-five residents were applied exam eligible, had passed all their Core Exams, but had not yet taken the Applied Exam. Seven residents passed the core exams they took but had not taken all four in series. Eight residents had outstanding failed Core Exams and therefore were not applied exam eligible. For the residents who met the inclusion criteria, the median Basic Exam total score was 82.3 with a minimum of 57.1 and a maximum of 95.5. The overall first-time pass percent rates for the Core Exams were 96% (1529 residents) for medical dermatology, 97% (1531 residents) for pediatric dermatology, 95% (1510 residents) for dermatopathology, and 97% (1540 residents) for surgical dermatology. For the Applied Exam, there was a first-time pass rate of 97% (1544 residents).

The Basic Exam score components were compared between the residents who passed and failed for each Core and Applied exam. For all the score components, the Basic Exam scores of the residents who passed the certification exam were higher, with a p-value of <0.001, than the residents who failed the certification exam, as seen in Table 1.

**Table 1:** Comparison of Basic Exam Scores Between Fail and Pass Groups of each of the Core and Applied Exams Mann-Whitney U test

Certification Exam	Basic Exam Score Variable	Fail (mean (SD))	Pass (mean (SD))	P-Value	U value
Medical Dermatology Core Exam	Total Score	73.12 (6.08)	81.99 (6.15)	<0.001	13002.00
	Visual recognition	28.70 (25.77)	56.57 (28.84)	<0.001	20131.50
	Medical Dermatology	19.55 (19.96)	54.59 (28.55)	<0.001	13770.00
	Pediatric Dermatology	26.00 (22.93)	57.33 (28.94)	<0.001	17444.50
	Dermatopathology	33.25 (27.96)	57.51 (28.85)	<0.001	22800.50
	Surgical Dermatology	37.32 (27.42)	57.58 (29.03)	<0.001	26332.00
	Science	35.30 (26.93)	56.07 (28.83)	<0.001	25521.50
Pediatric Dermatology Core Exam	Total Score	73.13 (5.79)	81.98 (6.17)	<0.001	12222.50
	Visual recognition	29.22 (24.45)	56.51 (28.91)	<0.001	19626.50
	Medical Dermatology	19.26 (19.10)	54.55 (28.58)	<0.001	13080.50
	Pediatric Dermatology	25.26 (22.19)	57.32 (28.95)	<0.001	16153.00
	Dermatopathology	32.24 (24.35)	57.52 (28.95)	<0.001	21114.50
	Surgical Dermatology	38.61 (28.51)	57.51 (29.03)	<0.001	26579.00
	Science	35.61 (29.11)	56.04 (28.77)	<0.001	25057.00
Dermatopathology Core Exam	Total Score	74.41 (6.09)	82.04 (6.16)	<0.001	20720.50
	Visual recognition	35.08 (25.71)	56.60 (28.98)	<0.001	32845.50
	Medical Dermatology	27.97 (24.42)	54.61 (28.66)	<0.001	27161.00
	Pediatric Dermatology	33.97 (26.40)	57.33 (29.02)	<0.001	30888.50
	Dermatopathology	26.11 (23.00)	58.17 (28.60)	<0.001	22543.50
	Surgical Dermatology	39.67 (27.85)	57.72 (29.02)	<0.001	36839.50
	Science	34.88 (25.99)	56.36 (28.78)	<0.001	33033.00
Surgical Dermatology Core Exam	Total Score	73.67 (5.89)	81.91 (6.22)	<0.001	11529.50
	Visual recognition	30.00 (26.63)	56.33 (28.93)	<0.001	17416.50
	Medical Dermatology	23.24 (21.44)	54.23 (28.75)	<0.001	13682.00
	Pediatric Dermatology	31.78 (23.22)	56.94 (29.18)	<0.001	17882.50
	Dermatopathology	31.89 (24.19)	57.38 (28.98)	<0.001	17157.00
	Surgical Dermatology	32.71 (28.95)	57.57 (28.92)	<0.001	18704.50
	Science	27.87 (23.05)	56.14 (28.78)	<0.001	15619.50
Applied Exam	Total Score	69.47 (5.16)	82.00 (6.06)	<0.001	3803.50
	Visual recognition	21.80 (18.06)	56.48 (28.89)	<0.001	10579.50
	Medical Dermatology	11.10 (9.60)	54.47 (28.52)	<0.001	5238.00
	Pediatric Dermatology	19.29 (17.79)	57.20 (28.93)	<0.001	8977.00
	Dermatopathology	19.90 (19.24)	57.63 (28.75)	<0.001	9243.50
	Surgical Dermatology	27.07 (20.35)	57.65 (29.00)	<0.001	13181.50
	Science	26.22 (22.33)	56.11 (28.77)	<0.001	13239.00

**Figure 1:**
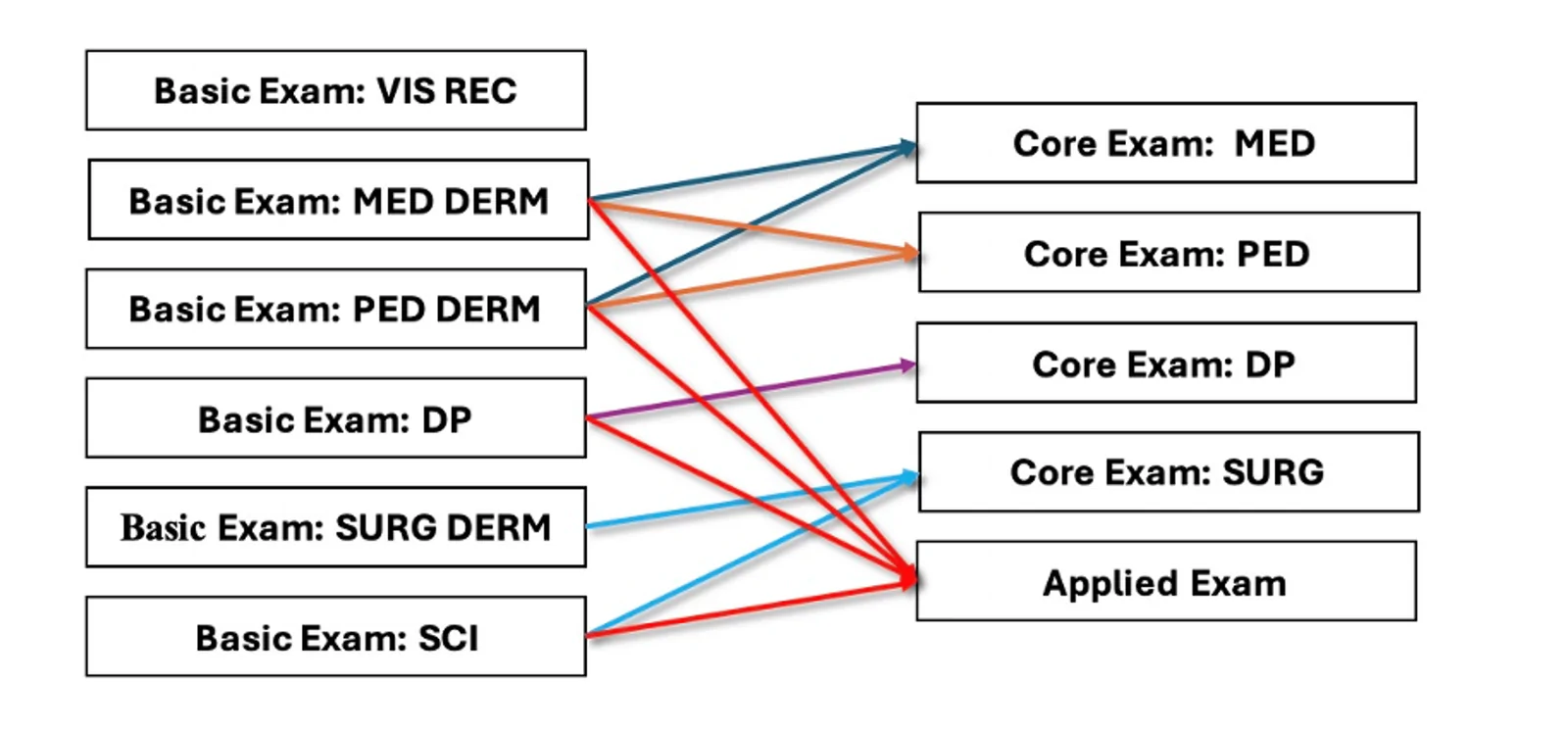
A path diagram of the statistically significant Basic Exam score components to Core/Applied Exam pass/fail outcome. Green arrow designates the statistically significant Basic Exam score components to Medical Dermatology Core Exam, orange arrow designates the statistically significant Basic Exam score components to Pediatric Dermatology Core Exam, purple arrow designates the statistically significant Basic Exam score components to Dermatopathology Core Exam, blue arrow designates the statistically significant Basic Exam score components to Surgical Dermatology Core Exam and red arrow designates the statistically significant Basic Exam score components to Applied Exam.

Multivariate logistic regression analysis was performed to further investigate the Basic Exam score components associated with pass/fail outcome on the certification exams with the results presented in Table 2. Figure 1 demonstrates which basic exam score components were significant to which Core or Applied Exam.

**Table 2:** Multivariate Logistic Regression Analysis Between Pass/Fail Outcome and the Basic Exam Components. OR: Odds ratio, 95% CI: 95% confidence interval

Core Exam	Basic Exam Variable	OR	P-value	95% CI
Medical Dermatology Core Exam	Visual recognition	0.9991	0.9077	(0.9835, 1.01559)
	Medical Dermatology	1.0393	0.0009	(1.01646, 1.06408)
	Pediatric Dermatology	1.0201	0.0043	(1.00678, 1.0347)
	Dermatopathology	1.0039	0.5313	(0.99204, 1.01636)
	Surgical Dermatology	1.0032	0.5667	(0.99248, 1.01436)
	Science	1.0077	0.1805	(0.99669, 1.01936)
Pediatric Dermatology Core Exam	Visual recognition	0.9957	0.6026	(0.98001, 1.01235)
	Medical Dermatology	1.0428	0.0005	(1.01929, 1.06846)
	Pediatric Dermatology	1.0217	0.0029	(1.00795, 1.03686)
	Dermatopathology	1.0062	0.3334	(0.99397, 1.01915)
	Surgical Dermatology	1.0004	0.9408	(0.98964, 1.01161)
	Science	1.0065	0.2644	(0.99536, 1.01827)
Dermatopathology Core Exam	Visual recognition	1.0017	0.8097	(0.98794, 1.01612)
	Medical Dermatology	1.0108	0.2208	(0.99372, 1.02847)
	Pediatric Dermatology	1.0088	0.1075	(0.9983, 1.01979)
	Dermatopathology	1.0306	9.63E-07	(1.01865, 1.0436)
	Surgical Dermatology	1.0025	0.6088	(0.99315, 1.01205)
	Science	1.0090	0.0716	(0.99939, 1.0192)
Surgical Dermatology Core Exam	Visual recognition	1.0092	0.3211	(0.99159, 1.02823)
	Medical Dermatology	1.0155	0.1948	(0.99252, 1.03992)
	Pediatric Dermatology	1.0087	0.2201	(0.99519, 1.02339)
	Dermatopathology	1.0061	0.3823	(0.99279, 1.02037)
	Surgical Dermatology	1.0129	0.0476	(1.00047, 1.02626)
	Science	1.0230	0.0014	(1.0094, 1.03813)
Applied Exam	Visual recognition	0.9953	0.6616	(0.97494, 1.01728)
	Medical Dermatology	1.0693	0.0006	(1.03147, 1.11445)
	Pediatric Dermatology	1.0244	0.0152	(1.00582, 1.04596)
	Dermatopathology	1.0220	0.0211	(1.00439, 1.04262)
	Surgical Dermatology	1.0125	0.0939	(0.99841, 1.02811)
	Science	1.0160	0.0402	(1.00137, 1.03246)

The optimal threshold for Basic Exam total score and percentile rank were then calculated using a receiver operating characteristic (ROC) curve to determine if there was a threshold above which passing a Core/Applied Exam was more likely to occur. The threshold total scores ranged from 75.55 to 79.55, and the threshold percentile ranks ranged from 22-38% for the different Core and Applied Exams, as seen in Table 3. The average total score of 78.5 and average percentile rank of 31.4% would capture 175 (77.8%) and 174 (77.3%) of 225 residents who failed the exams, respectively.

**Table 3:** Optimal threshold Cutoff scores for the Basic Exam total score and percentile rank PPV: positive predictive value; LR+: likelihood ratio for a positive test; AUC: area under curve; 95% CI: 95% confidence interval

Measure	Medical Dermatology Core Exam	Pediatric Dermatology Core Exam	Dermatopathology Core Exam	Surgical Dermatology Core Exam	Applied Exam
Cutoffs of Basic Exam Total Score					
Threshold	79.55	78.55	79.55	79.05	75.55
Sensitivity	0.68	0.73	0.69	0.70	0.85
Specificity	0.89	0.83	0.83	0.84	0.93
PPV	99.59%	99.40%	99.60%	99.50%	98.84%
LR+	6.38	4.381	3.969	4.53	11.63
AUC (95% CI)	0.85(0.80-0.89)	0.85(0.81-0.89)	0.82(0.77-0.86)	0.83(0.79-0.88)	0.94(0.92-0.96)
Cutoffs of Basic Exam Percentile Rank					
Threshold	34	29	38	34	22
Sensitivity	0.7188	0.7622	0.69	0.71	0.84
Specificity	0.875	0.8148	0.87	0.87	0.95
PPV	98.45%	98.35%	98.67%	98.45%	98.14%
LR+	5.75	4.116	5.17	5.36	17.13
AUC (95%CI)	0.86(0.81-0.90)	0.86(0.82-0.90)	0.82(0.78-0.87)	0.84(0.80-0.89)	0.94(0.92-0.96)

Kruskal-Wallis test was performed to see if there were any differences in the total scores of the basic exam among residents from different programs. There are 149 programs in the data set. A p-value < 0.001 was found, but given the large number of programs and small number of residents in some of the programs, this p-value may not be reliable or meaningful.

## Discussion

This is the first study to demonstrate a significant and direct correlation between the new Basic Exam scores and outcomes in the ABD initial certification pathway. The Basic Exam is intended to assess first-year dermatology residents’ foundation of knowledge in dermatology at the beginning of residency. Until now, it has been unclear how residents, program directors, and faculty mentors should interpret these results to help residents prepare for the new certification exam pathway that now begins in the second year of residency. To become a board-certified dermatologist, residents must successfully pass all four Core Exams and then the Applied Exam.

Previous studies in other medical specialties have shown that self-assessment ITE exams correlate with end-of-residency certification exam pass rate [1-3, 9]. The USMLE Step performance had also been correlated with outcomes on the ITE exams in dermatology [10]. As USMLE Step 1 is now pass-fail and USMLE Step 2 is not mandatory to apply to most dermatology programs [11], dermatology may shift to prioritization of other applicant characteristics in the selection process, and the test-taking achievement of its residents may be more variable. This study confirms that the Basic Exam scores are a useful tool in early recognition of residents that struggle with medical knowledge or test-taking abilities.

This study confirmed that most dermatology residents will be successful on their certification exams, with the certification exam pass rate varying between 95-97% on the first attempt. For residents with focused areas of medical knowledge deficits, the individual basic exam score components can be helpful in informing a resident which Core certification exams they may struggle with. The multivariant logistic analysis found that the medical and pediatric dermatology Basic Exam score components are significantly associated with the Medical and Pediatric Core Exams. The dermatopathology Basic Exam score was associated with Dermatopathology Core Exam performance. The basic science and surgical dermatology Basic Exam score components were associated with Surgical Core Exam outcomes. Since residents can choose when and in which order to take the Core Exams during their second and third year of residency, they may want to take those that correlate with areas of lower scores as a later exam or as a single exam in a testing block to allow them time to focus on that material prior to their certification exam.

The threshold scores for total Basic Exam score and percentile rank can help residents and program directors know who is at greatest overall risk of certification exam failure. The threshold cutoff varied by certification exam, with a total exam score range of 75.55 to 79.55 and percentile rank range of 22%-38%. While many of the residents below these ranges will not experience certification failure, as it encompasses the bottom third of all resident scores, this may be a yellow flag for the residents and program directors to assess an individual resident’s study strategies and offer additional support if needed if they fall below these cutoff points. This strategy would capture most residents who experience certification exam failures, as 77% of residents who failed the exam fell below the average threshold of a total score of 78.5 and average percentile rank of 31.4%.

This study is limited in that only residents who took all exams were included in the analysis. However, only eight residents in the dataset had outstanding Core Exam failures that would have prevented them from sitting for the applied exam, so its effect on the overall data is likely small. Another limitation is that the ABD does not collect demographic data on dermatology residents. Therefore, analysis of resident characteristics that also influenced their performance on the Basic Exam and certification exams could not be performed. The residency program was analyzed as an independent factor in the resident’s performance on the Basic Exam and was found to be significant, but given the large number of programs and small numbers of individual residents in some programs, this analysis may not be meaningful. This could be an area for future exploration.

## Conclusions

This study of the first three dermatology residency classes to take the new self-assessment and certification exam pathway demonstrates that the self-assessment Basic Exam is a meaningful marker of residents’ future initial certification exam performance, and that a review of the total score, percentile rank and score components may provide residents, program directors and faculty mentors data that can help identify or confirm concerns about a resident’s medical knowledge or test taking ability. Future studies are needed to determine the best educational strategies for residents and residency programs with low basic exam scores.

## References

[1] Girotto JA, Adams NS, Janis JE, Brandt KE, Slezak SS (2019). Performance on the plastic surgery in-service examination can predict success on the American Board of Plastic Surgery written examination. Plast Reconstr Surg.

[2] Puscas L (2019). Junior otolaryngology resident in-service exams predict written board exam passage. Laryngoscope.

[3] Rayamajhi S, Dhakal P, Wang L, Rai MP, Shrotriya S (2020). Do USMLE steps, and ITE score predict the American Board of Internal Medicine Certifying Exam results?. BMC Med Educ.

[4] Tarras S, White MT, Toloff K, Cooley D, Edelman D (2022). Just do it: participation in structured online curricula reliably improves low ABSITE scores. J Surg Educ.

[5] Borman KR (2006). Does academic intervention impact ABS qualifying examination results?. Curr Surg.

[6] (2025). American Board of Dermatology. ABD Certification Pathway Info Center. https://www.abderm.org/residents-and-fellows/abd-certification-pathway/abd-certification-pathway-info-center.

[7] (2025). American Board of Dermatology. ABD Certification Pathway Exam Pass Rates. https://www.abderm.org/residents-and-fellows/abd-certification-pathway/abd-certification-pathway-exam-pass-rates.

[8] (2025). American Council for Graduate Medical Education. ACGME Program Requirements for Graduate Medical Education in Dermatology. https://www.acgme.org/globalassets/pfassets/programrequirements/080_dermatology_2023.pdf.

[9] McDonald FS, Jurich D, Duhigg LM (2020). Correlations between the USMLE Step Examinations, American College of Physicians In-Training Examination, and ABIM Internal Medicine Certification Examination. Acad Med.

[10] Fening K, Vander Horst A, Zirwas M (2011). Correlation of USMLE Step 1 scores with performance on dermatology in-training examinations. J Am Acad Dermatol.

[11] (2025). National Resident Matching Program. Charting Outcomes: Program Director Survey Results, 2024 Main Residency Match. https://www.nrmp.org/match-data/2024/08/charting-outcomes-program-director-survey-results-main-residency-match.

